# A LASSO-based nomogram for predicting sarcopenia-related nutritional risk in esophageal cancer patients: model development, validation, and an interactive clinical decision tool

**DOI:** 10.3389/fonc.2026.1863123

**Published:** 2026-09-01

**Authors:** Jiwei Zhang, Yayun Cui, Zhen Zong, Wei Zhang

**Affiliations:** Taikang Xianlin Drum Tower Hospital, Affiliated Hospital of Medical School, Nanjing University, Nanjing, China

**Keywords:** esophageal cancer, interpretability, predictive model, sarcopenia, SHAP

## Abstract

**Objective:**

This study aims to construct an interpretable predictive model for esophageal cancer sarcopenia-related nutritional risk based on multidimensional clinical indicators and develop an interactive clinical decision support system.

**Methods:**

The study cohort comprised 202 patients with a confirmed diagnosis of esophageal cancer who were retrospectively recruited from Taikang Xianlin Drum Tower Hospital. The study period spanned from January 2023 to December 2025. The sample was split into training and validation datasets (7:3), respectively. Sarcopenia nutritional risk was defined as NRS-2002 score ≥3. Predictors were screened using LLogistic and LASSO regression to construct a model. Model performance was evaluated using ROC, calibration, decision curve analysis (DCA), and CIC curves. Model interpretability was analyzed using SHAP values. An interactive online prediction system was developed based on R Shiny.

**Results:**

Age, sex, BMI, diabetes, intervention, prothrombin time (PT), and clinical stage (cStage) were predictors of sarcopenia-related nutritional risk. The AUC was 0.864 (0.778–0.940) in the training set and 0.802 (0.696–0.898) in the validation set. The calibration curves showed satisfactory goodness of fit (*P* > 0.05), and DCA indicated clinical net benefit within the threshold range of 0.05–0.70. SHAP analysis revealed that BMI, PT, and gender were key contributing factors. The interactive prediction system “Sarcopenia Intelligence” was successfully developed (https://ytky622.shinyapps.io/Sarcopenia_Predictor_2026/).

**Conclusion:**

The model demonstrates favorable discrimination, calibration, and clinical utility. Combined with SHAP interpretability and the interactive system, it provides a convenient and precise tool for early screening and individualized intervention of sarcopenia in patients with esophageal cancer.

## 1 Introduction

Sarcopenia manifests as a systemic syndrome, in which skeletal muscle mass and function deteriorate progressively, and disproportionately affects patients with esophageal malignancy. Published reports indicate that its prevalence in this population reaches 46.3% (range, 14.4%–81%), which significantly elevates the risks of postoperative complications, chemotherapy-related toxicity, and mortality in this patient population (1, 2). In recent years, the deleterious effect of sarcopenia on the treatment effectiveness of neoadjuvant immunochemotherapy for esophageal cancer has received increasing attention. Studies have shown that excessive skeletal muscle loss during treatment maintains an independent association with survival outcomes among esophageal cancer patients (3). Currently, sarcopenia diagnosis primarily relies on skeletal muscle index derived from computed tomography imaging, which necessitates specialized software for cross-sectional plane delineation. This methodology is operationally cumbersome and constrained by single time-point assessment and inadequate evaluation of dynamic changes (4, 5). Furthermore, existing investigations have predominantly focused on unimodal imaging data, neglecting the integration of multidimensional clinical information encompassing nutritional metabolism, inflammatory status, and tumor characteristics. Machine learning techniques have demonstrated substantial advantages in sarcopenia risk prediction. Recent studies have employed algorithms such as random forest and XGBoost to construct sarcopenia prediction models for community populations, achieving AUC values of 0.85–0.999, and realized model interpretability using SHAP analysis (5, 6). However, studies on sarcopenia predictive models specifically for esophageal cancer are scarce. Moreover, the existing models are mostly static nomograms, which lack interactive dynamic prediction, individualized prediction, and interpretable analysis (7). However, current evidence linking sarcopenia to esophageal cancer outcomes remains largely confined to single cross-sectional analyses. Multi-dimensional predictive frameworks that integrate clinical baseline information, nutritional indicators, and metabolic indicators lead to deficiencies such as delayed identification of high-risk patients and delayed intervention timing (8).

Therefore, this study aims to integrate multi-dimensional data to construct an interpretable predictive model of sarcopenia-related nutritional risk in esophageal cancer and develop an interactive clinical decision support system. By integrating multi-dimensional information such as demographic characteristics, tumor staging, laboratory parameters, and comorbidities, an individualized sarcopenia predictive model for esophageal cancer is intended to be constructed. This model is intended to assist clinicians in identifying patients who need early dietitian referral, rehabilitation assessment, enteral feeding evaluation, or confirmatory body composition testing before treatment intolerance develops.

## 2 Methods

### 2.1 Study subjects

We retrospectively included 202 consecutive patients with esophageal cancer who received treatment at the oncology department of Taikang Xianlin Drum Tower Hospital between January 2023 and December 2025.

**Figure 1:**
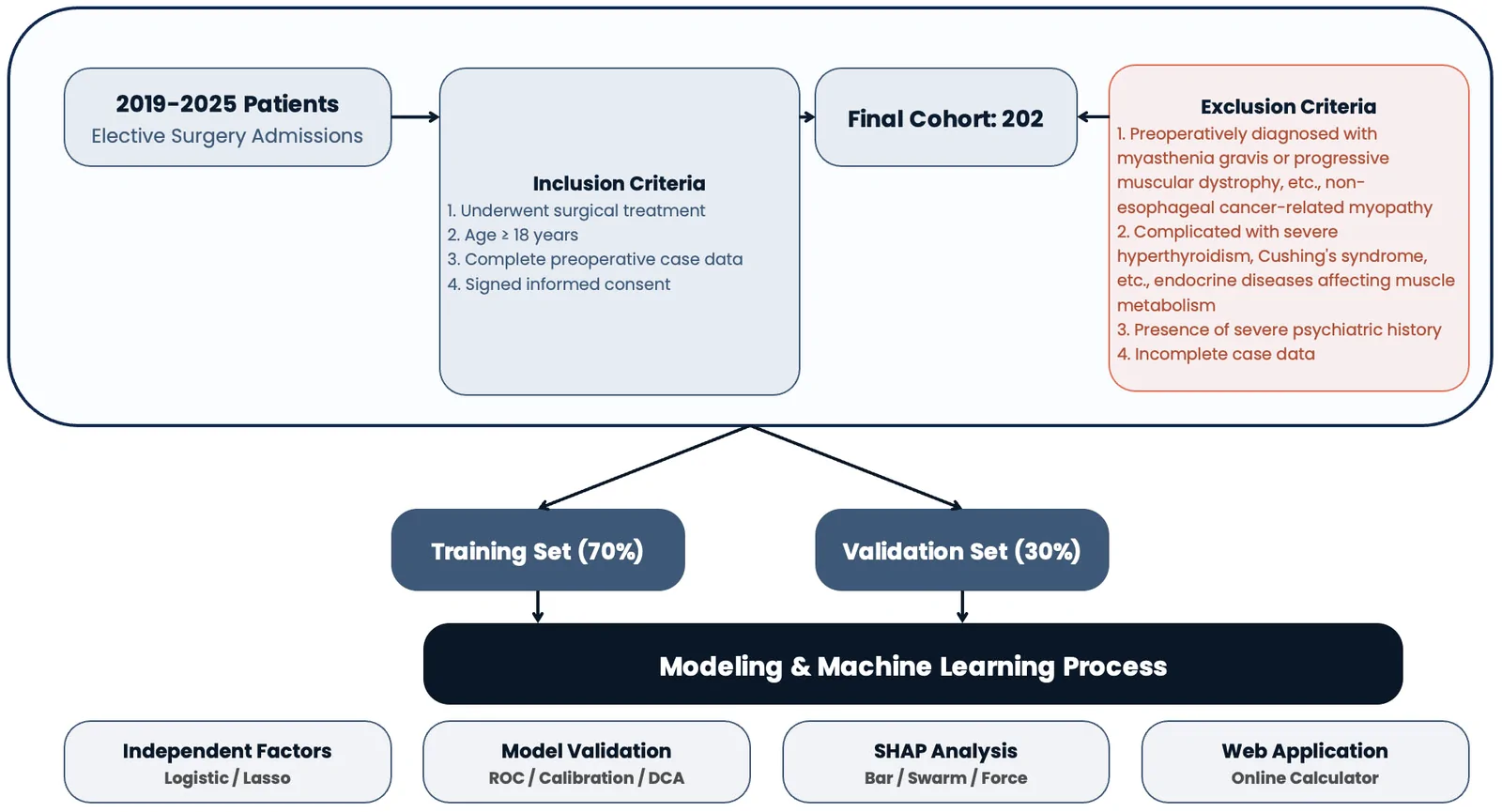
Technical roadmap.

The inclusion criteria were as follows: (1) histopathologically confirmed diagnosis of esophageal cancer, (2) age ≥18 years, and (3) complete clinical data, including demographic information, laboratory examinations, imaging data, and pathological results. Meanwhile, the exclusion criteria include the following: (1) preoperative diagnosis of non-esophageal cancer-related myopathies, such as myasthenia gravis and progressive muscular dystrophy, (2) comorbidity with severe endocrine disorders affecting muscle metabolism, including hyperthyroidism and Cushing’s syndrome, (3) presence of a history of severe psychiatric disorders, and (4) incomplete clinical data. A retrospective cohort study design was employed in this study. All patients with esophageal cancer were randomly assigned to the training and validation cohorts (7:3), respectively, for the development and validation of the predictive model. The training cohort was used for model development and parameter estimation, while the internal validation cohort was applied to evaluate the generalization ability of the model. This study was approved by the Medical Ethics Committee of Taikang Xianlin Drum Tower Hospital (ethics approval no.: 2026-001-01) (**Figure 1**). This predictive model study was reported in accordance with the TRIPOD guidelines.

### 2.2 Diagnostic criteria for sarcopenia-related nutritional risk

The diagnosis of sarcopenia was established in accordance with the *Asian Working Group for Sarcopenia 2025 Consensus* (*AWGS 2025*) (9). As this was a retrospective study, direct measurements of muscle strength, physical performance, and CT-based skeletal muscle were not available. The Nutritional Risk Screening 2002 (NRS-2002) score was used as a surrogate indicator for assessing sarcopenia risk. Yao J et al. (10) confirmed that the NRS-2002 score was significantly correlated with skeletal muscle index measured by computed tomography (CT). Moreover, an NRS-2002 score ≥3 was independently associated with reduced skeletal muscle density; Hanna-Deschamps E et al. (11) further confirmed that the prevalence of sarcopenia reached 90.3% among elderly hospitalized patients with an NRS-2002 score ≥3, and this factor remained independently linked to sarcopenia. In the study, an NRS-2002 score ≥3 was defined as having high sarcopenia-related nutritional risk, while a score <3 was defined as low risk.

### 2.3 Predictors

#### 2.3.1 Collection of baseline characteristics

The following data were collected by dedicated researchers at patient admission using standardized questionnaires and the electronic medical record system: (1) demographic characteristics: age, sex, BMI, smoking history, and drinking history, (2) medical history and comorbidities: history of diabetes mellitus, hypertension, liver disease, coronary heart disease (CHD), and dysphagia, (3) tumor-related characteristics: tumor location (upper, middle, or lower thoracic esophagus), tumor size (maximum diameter), histology, differentiation degree, metastatic lymph node status (Met_Lymph), vascular invasion status, and clinical stage (cStage, with subclassification of cT stage [cTT3– cTT4], cM stage [cMM1], and cStage IV), and (4) treatment-related information: surgery, receipt of neoadjuvant therapy (yes/no), receipt of immunotherapy, receipt of targeted therapy, receipt of palliative treatment, and placement of a nutritional tube (nasogastric tube or gastrostomy tube).

#### 2.3.2 Laboratory examinations

Early-morning venous sampling was performed upon admission. Comprehensive panels assessed the following: (1) hematologic indices (WBC, RBC, hemoglobin, platelets, and MONO), (2) hepatic enzymes (ALT, AST, and γ-GT) and bilirubin metabolism (DBIL), (3) BUN, (4) PT, APTT, INR, fibrinogen, and D-dimer (DD), (5) inflammatory status (CRP), (6) tumor markers (CEA, CA19-9, CA125, CA242, CA72-4, SCC, and AFP), (7) hepatic fibrosis via FIB-4 calculation, and (8) metabolic and muscular enzymes (GLU, CK, and CKMB).

### 2.4 Statistical analysis

Statistical analyses were conducted in R (version 4.3.1). A total of 202 patients were randomly assigned to the training and validation cohorts (7:3), respectively. Random reproducibility was ensured by setting a random seed (set.seed (123)). Predictor selection combined established literature and clinical expertise with univariate (*P* < 0.10) and multivariate (*P* < 0.05) logistic regression, followed by LASSO regression with 10-fold cross-validation to optimize λ (minimum error at log(λ) = −3.45). Variables with >5% missing values were excluded; the remaining missing values were imputed using multiple imputation by chained equations (MICE, 10 iterations). Multicollinearity was assessed via variance inflation factor (VIF), with all final predictors showing VIF <2.5. Measurement data were expressed as median (IQR) or mean ± SD. Between-group comparisons were performed using Student’s *t*-test. Categorical data were presented as *n* (%), with between-group comparisons analyzed using chi-square test. Univariate logistic regression analysis (*P* < 0.10) was performed to screen potential predictors. Subsequently, multivariate logistic regression (*P* < 0.05) was conducted to identify independent predictors. To further optimize the predictor combination and reduce dimensionality, LASSO regression was then applied to the candidate variables retained from the univariate analysis. Finally, considering clinical practicality and the availability of routine bedside indicators, the final predictors were determined and incorporated into the predictive model. We assessed discriminative ability with ROC analysis, predictive accuracy with calibration curves, and clinical net benefit with decision curve analysis. The SHAP method was applied to the final logistic regression model using KernelSHAP, based on the fitted coefficients, to interpret the model, and subgroup analysis stratified by seven indicators was performed to validate the generalization ability. An interactive prediction system was developed using R Shiny (version 1.8.0). *P* < 0.05 was considered statistically significant.

## 3 Result

### 3.1 Comparison of baseline characteristics

There were no statistically significant differences in baseline characteristics between the two groups, including age, sex, BMI, tumor size, routine blood parameters, liver and renal function, tumor markers, comorbidities, and treatment modalities (all *P >*0.05) (**Table 1**).

**Table 1:** Baseline characteristics.

Variable, *n*	Level	Training, 141	Validation, 61	*p*
Age, median (IQR)		73.00 [68.00, 79.00]	71.00 [60.75, 76.75]	0.052
Sex, *n* (%)	Female	34 (24.1)	11 (18.0)	0.442
	Male	107 (75.9)	50 (82.0)	
BMI, mean (SD)		20.72 (2.94)	20.78 (3.79)	0.906
Smoking, *n* (%)	No	130 (92.2)	50 (82.0)	0.050
	Yes	11 (7.8)	11 (18.0)	
Size, median (IQR)		3.10 [2.70, 3.60]	3.10 [2.60, 3.70]	0.411
WBC, median (IQR)		5.05 [3.86, 6.76]	5.31 [3.91, 7.89]	0.345
RBC, median (IQR)		3.90 [3.42, 4.25]	3.81 [3.31, 4.23]	0.388
HGB, mean (SD)		116.98 (18.17)	114.25 (20.50)	0.347
PLT, median (IQR)		183.00 [136.00, 242.00]	188.00 [154.00, 249.00]	0.250
CRP, median (IQR)		12.84 [6.85, 23.76]	13.10 [6.20, 31.89]	0.970
ALT, median (IQR)		15.00 [11.00, 24.70]	15.00 [11.00, 23.00]	0.674
AST, median (IQR)		20.00 [17.00, 26.00]	18.00 [15.40, 23.00]	0.054
FIB4, median (IQR)		2.00 [1.60, 3.10]	2.00 [1.30, 2.50]	0.069
CA125, median (IQR)		18.13 [8.79, 38.70]	19.30 [10.20, 67.24]	0.385
CA199, median (IQR)		11.10 [7.08, 20.89]	11.90 [6.73, 20.20]	0.757
AFP, median (IQR)		2.75 [2.14, 3.24]	3.00 [2.16, 3.40]	0.270
CEA, median (IQR)		2.75 [1.58, 5.07]	2.72 [1.63, 3.84]	0.785
CA242, median (IQR)		7.71 [4.31, 11.62]	8.05 [3.52, 13.40]	0.855
CA724, median (IQR)		2.09 [0.65, 6.64]	2.70 [0.68, 6.76]	0.525
SCC, median (IQR)		2.41 [1.13, 5.25]	2.08 [0.88, 4.54]	0.490
Intervention, *n* (%)	No	86 (61.0)	38 (62.3)	0.986
	Yes	55 (39.0)	23 (37.7)	
Radical CRT, *n* (%)	No	67 (47.5)	31 (50.8)	0.781
	Yes	74 (52.5)	30 (49.2)	
Immunotherapy, *n* (%)	No	59 (41.8)	26 (42.6)	1.000
	Yes	82 (58.2)	35 (57.4)	
Targeted therapy, *n* (%)	No	119 (84.4)	55 (90.2)	0.386
	Yes	22 (15.6)	6 (9.8)	
Palliative, *n* (%)	No	131 (92.9)	55 (90.2)	0.705
	Yes	10 (7.1)	6 (9.8)	
Catheterization, *n* (%)	No	109 (77.3)	43 (70.5)	0.394
	Yes	32 (22.7)	18 (29.5)	
Diabetes, *n* (%)	No	124 (87.9)	54 (88.5)	1.000
	Yes	17 (12.1)	7 (11.5)	
Hypertension, *n* (%)	No	84 (59.6)	34 (55.7)	0.724
	Yes	57 (40.4)	27 (44.3)	
COPD, *n* (%)	No	136 (96.5)	59 (96.7)	1.000
	High	32 (22.7)	15 (24.5)	
Sarcopenia-related nutritional risk	Low	109 (77.3)	46 (75.4)	0.769

### 3.2 Logistic regression analysis of sarcopenia-related nutritional risk-associated factors

We first applied univariate logistic regression to screen candidate variables for sarcopenia in esophageal cancer. Those with a *P*-value <0.10 were subsequently included in a multivariate model. After adjustment, age (OR = 1.10) and PT (OR = 1.59) remained independently associated with sarcopenia (**Table 2**).

**Table 2:** Logistic regression analysis of sarcopenia-associated factors.

Variable	OR	Lower_CI	Upper_CI	*P*-value
Liver disease	3.59	0.11	72.14	0.431
Age	1.10	1.00	1.22	0.030
AFP	1.26	0.71	2.15	0.401
GGT	1.00	1.00	1.00	0.295
CK	1.00	0.99	1.02	0.713
PT	1.59	1.07	2.72	0.024

AFP, alpha-fetoprotein; GGT, gamma-glutamyl transferase; CK, creatine kinase.

### 3.3 LASSO regression for predictor selection

**Figure 2:**
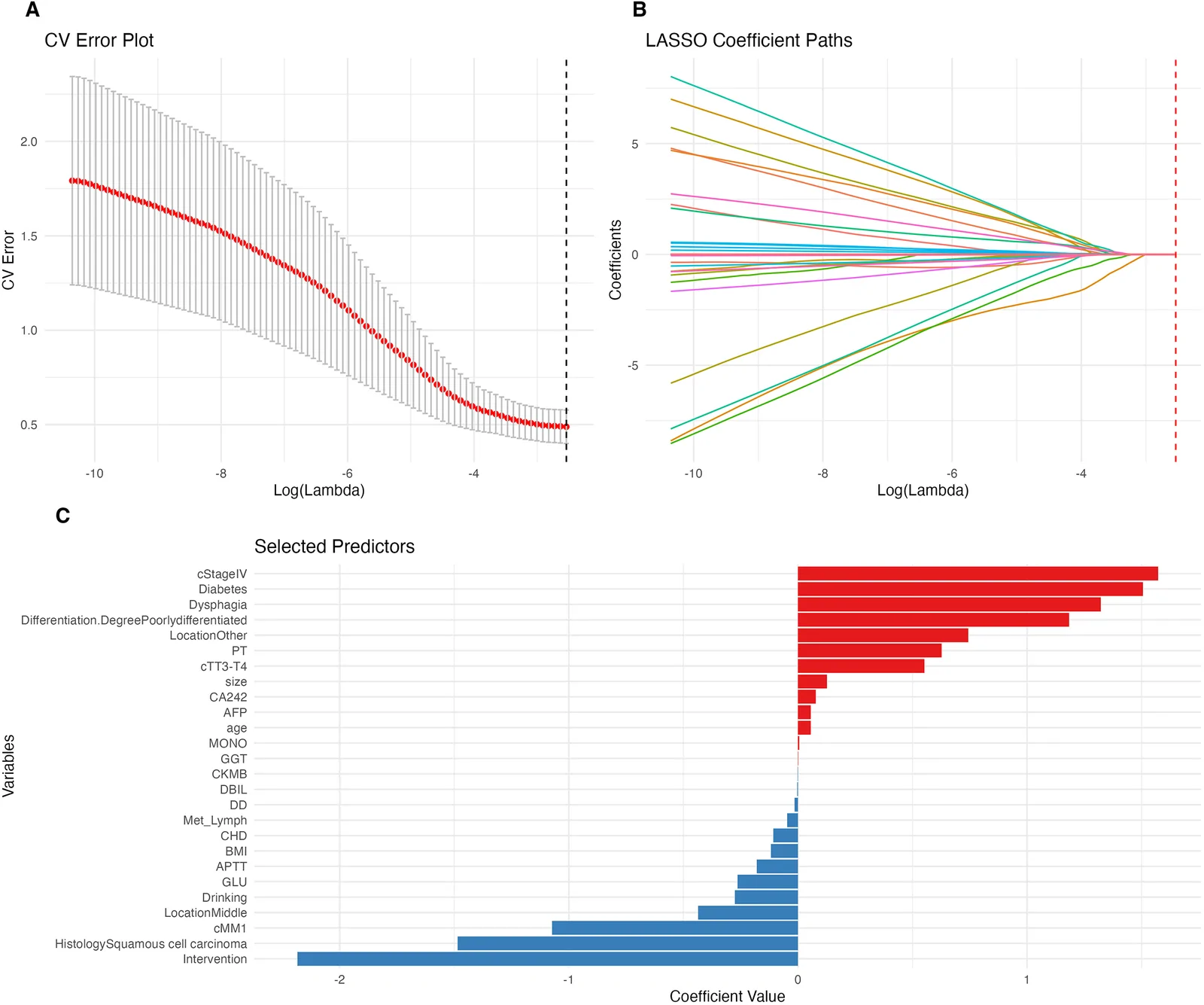
Key predictor selection based on LASSO regression. **(A)** 10-fold cross-validation error curve, **(B)** LASSO coefficient paths, and **(C)** predictors ranked by effect magnitude.

To further optimize the predictor combination, LASSO regression was employed in this study to reduce dimensionality and screen multidimensional indicators. Thus, 10-fold cross-validation was performed to select the optimal regularization parameter λ, with the minimum cross-validation error achieved at log(λ) = −3.45 (**Figure 2A**). The LASSO coefficient path plot demonstrated that the coefficients of each variable gradually shrank to zero as the λ value increased (**Figure 2B**). Ultimately, the five most influential and clinically readily available predictors were retained and incorporated into the subsequent model. Ranked by absolute coefficient value, these variables were as follows: cStage, treatment intervention, diabetes mellitus status, patient age, and PT (**Figure 2C**; **Table 3**).

**Table 3:** LASSO-selected predictor results.

Variable	Coefficient
Location: middle	-0.4361223
Location: other	0.74324557
Differentiation degree: poorly differentiated	1.18310708
Intervention	-2.1848467
Diabetes	1.50605353
CHD	-0.1071811
Drinking	-0.2746489
Dysphagia	1.32151222
Met_Lymph	-0.0472754
Histology: squamous cell carcinoma	-1.4849252
cTT3-T4	0.55220608
cMM1	-1.0728097
cStageIV	1.571396
BMI	-0.117057
Age	0.0557231
Size	0.12589782
AFP	0.05657246
CA242	0.07863041
MONO	0.00615246
GGT	0.00166035
DBIL	-0.0024489
GLU	-0.2632426
CKMB	-0.0013021
PT	0.62660601
APTT	-0.1799405
DD	-0.0142849

### 3.4 Development of nomogram and dynamic prediction system

**Figure 3:**
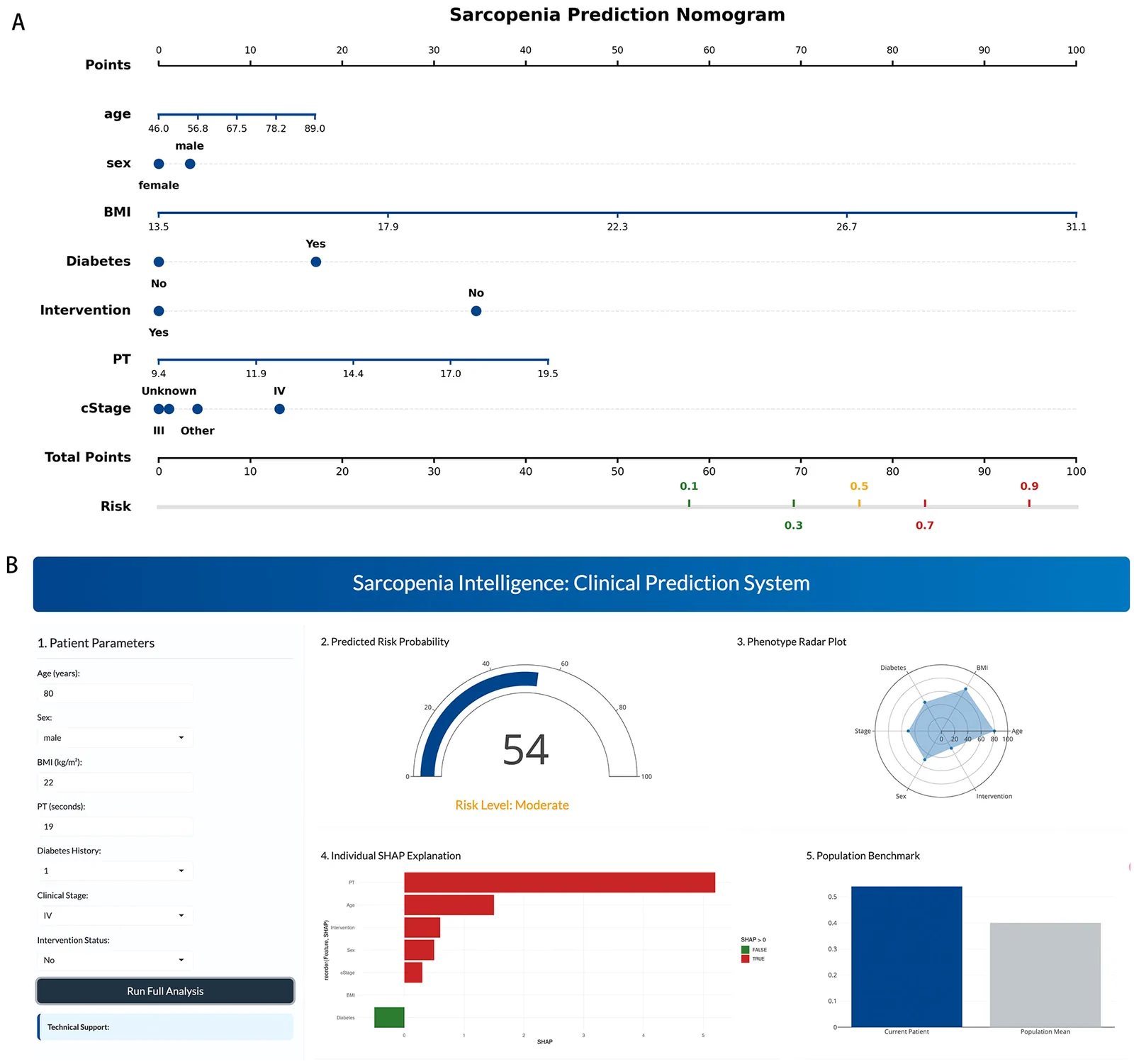
Nomogram and dynamic prediction system for sarcopenia risk in esophageal cancer patients. **(A)** Sarcopenia risk prediction nomogram. **(B)** Interactive dynamic prediction and interpretation system.

The final predictors were selected through a three-stage hybrid strategy integrating statistical evidence, clinical consensus, and operational feasibility, namely, stage 1: Age and PT were retained as they emerged as independently significant in multivariate logistic regression (*P* = 0.030 and *P* = 0.024) and were further supported by LASSO; stage 2: DM and cStage exhibited non-zero LASSO coefficients and are well-established clinically relevant factors for sarcopenia-related nutritional risk; intervention showed a substantial LASSO coefficient (−2.18) and reflects treatment intensity and disease severity that directly impacts nutritional status and muscle catabolism; and stage 3: Sex and BMI were incorporated from the univariate candidate pool based on biological plausibility and international consensus rather than purely statistical dominance—AWGS 2025 explicitly mandates sex-specific diagnostic cutoffs for sarcopenia, and BMI remains the most universally accessible nutritional indicator at bedside, particularly critical for detecting sarcopenic obesity where muscle loss may be masked by normal or high body weight. Given their clinical practicality and ease of bedside acquisition, these seven predictors were ultimately incorporated into the final nomogram as follows: age, sex, BMI, PT, diabetes, intervention, and cStage (**Figure 3A**). To enhance clinical utility, we developed an interactive dynamic prediction system named “Sarcopenia Intelligence: Clinical Prediction and Interpretation System” (**Figure 3B**: https://ytky622.shinyapps.io/Sarcopenia_Predictor_2026/). This system integrates five functional modules—namely, patient parameter input, real-time risk probability prediction, feature importance radar chart, individualized SHAP interpretation, and subgroup benchmark comparison—enabling the transformation of the predictive model from static visualization to dynamic, interpretable, and interactive clinical application.

### 3.5 Model performance evaluation

**Figure 4:**
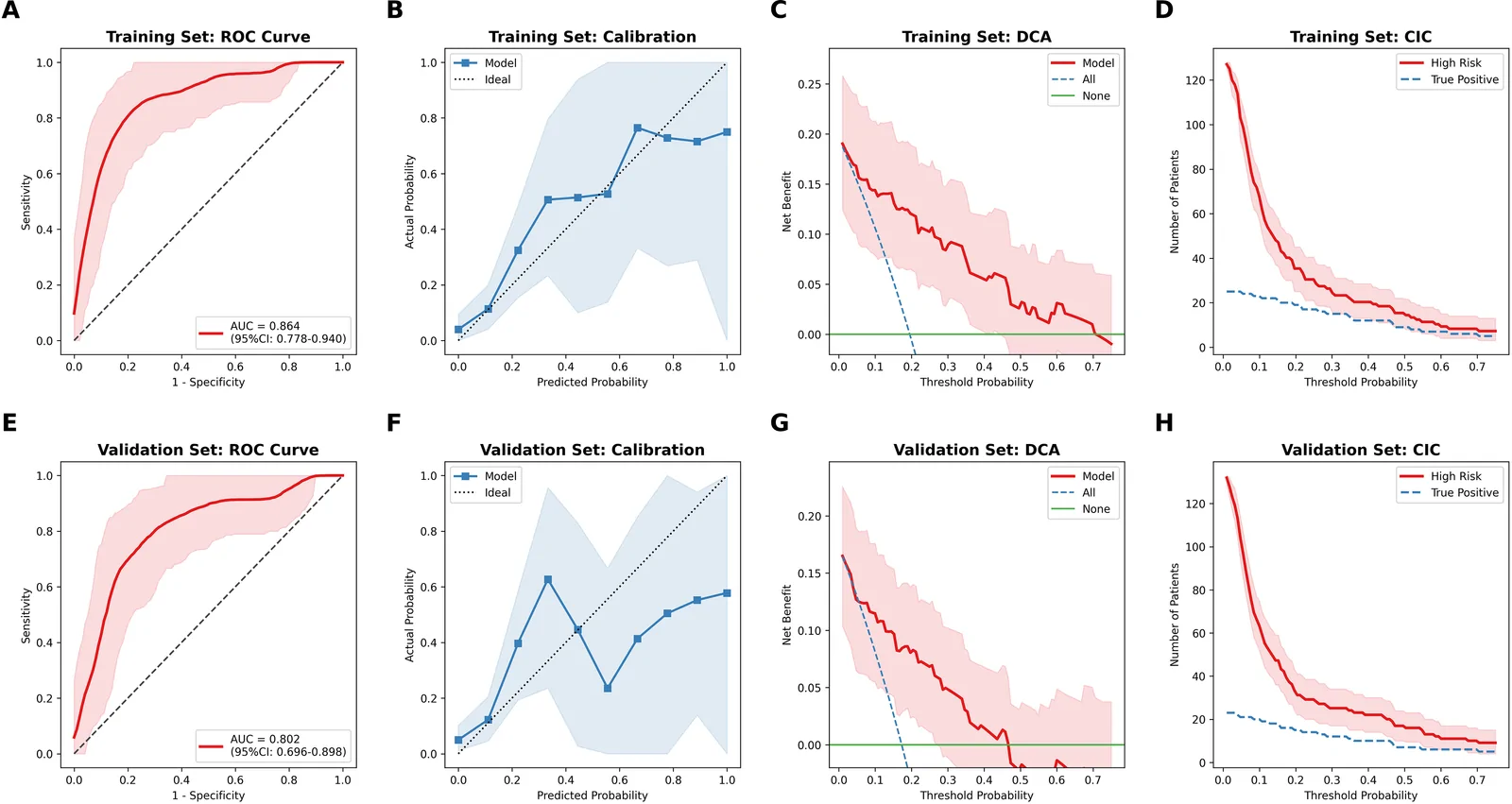
Performance evaluation of the sarcopenia prediction model. **(A, E)** ROC curve, **(B, F)** calibration curve, **(C, G)** DCA, and **(D, H)** CIC curve.

The AUC of the training set was 0.864 (CI: 0.778–0.940), and that of the validation set was 0.802 (CI: 0.696–0.898) (**Figures 4A, E**), indicating that the model had robust discrimination ability. Calibration curves revealed favorable consistency between predicted and actual risks of sarcopenia (**Figures 4B, F**). The decision curve analysis showed that the model yielded a positive net benefit across threshold probabilities from 0.05 to 0.70 (**Figures 4C, G**). Furthermore, the clinical impact curves (CICs) showed that the number of patients identified as high risk closely approximated the number of true-positive cases across clinically relevant threshold probabilities, supporting the potential clinical utility of the model (**Figures 4D, H**). LASSO regression with L1 regularization was used to mitigate overfitting. Bootstrap internal validation with 1,000 iterations yielded an optimism-corrected AUC of 0.839, a calibration slope of 0.94, a calibration intercept of −0.02, and a Brier score of 0.19. Collectively, these findings indicate that the model can effectively identify patients at high risk of sarcopenia and provide reliable evidence for clinical intervention decision-making (**Table 4**).

**Table 4:** Performance metrics of the sarcopenia prediction model.

Dataset	Auc	Acc	Sen	Spe	Youden
Training set	0.864 (0.778–0.940)	0.887 (0.681–0.965)	0.823 (0.500–1.000)	0.891 (0.664–0.970)	0.714 (0.425–0.948)
Validation set	0.802 (0.696–0.898)	0.882 (0.683–0.946)	0.764 (0.500–1.000)	0.890 (0.675–0.957)	0.654 (0.385–0.892)
Bootstrap (1,000×)	0.839 (0.805–0.873)	0.871 (0.742–0.938)	0.795 (0.523–0.958)	0.883 (0.701–0.954)	0.678 (0.412–0.912)

### 3.6 SHAP interpretability analysis of the prediction model

**Figure 5:**
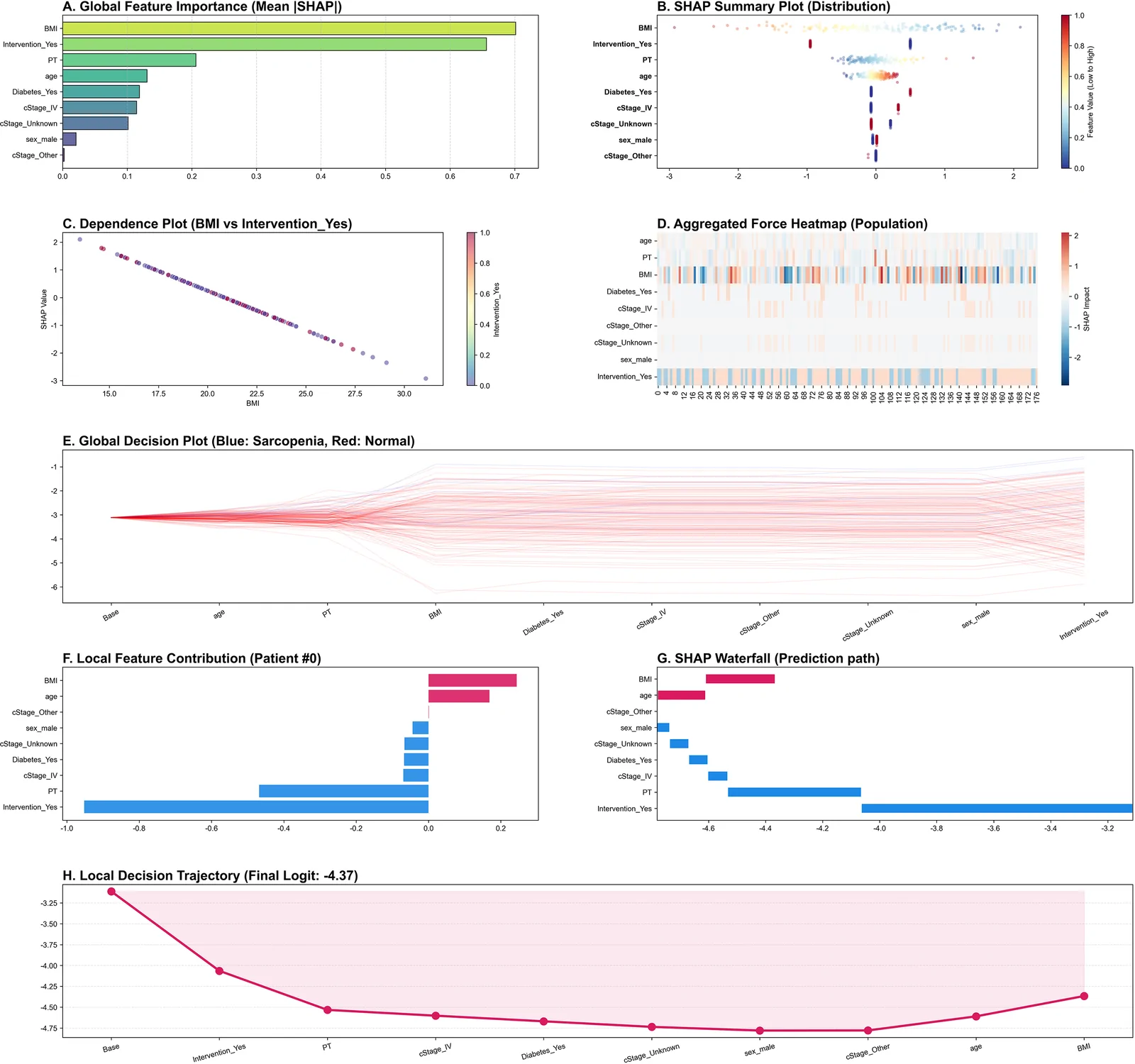
SHAP analysis of the sarcopenia prediction model. **(A)** Global feature importance is ranked. **(B)** SHAP summary plot (red: high-risk direction, blue: low-risk direction). **(C)** Dependence plot showing BMI–intervention interaction. **(D)** Aggregated force heatmap of population-level SHAP values. **(E)** Global decision plot (blue: sarcopenia patients, red: normal individuals). **(F)** Local feature contribution plot for patient #0. **(G)** SHAP waterfall plot for patient #0. **(H)** Local decision trajectory plot for patient #0 (final logit: -3.74).

We employed SHAP (SHapley Additive exPlanations) values to elucidate the contribution of individual predictors, revealing the contribution patterns of key predictors at both global and individual levels (**Figure 5**). The complete SHAP values (mean |SHAP|) were as follows: BMI (0.71), Intervention_Yes (0.25), PT (0.23), sex_male (0.18), cStage_Unknown (0.15), Diabetes_Yes (0.14), age (0.12), cStage_IV (0.11), and cStage_Other (0.09). Global feature importance analysis (**Figure 5A**) showed that BMI, intervention, and PT were the top three predictors driving model decisions, with the highest mean absolute SHAP values reflecting instance-level contributions including interactions, whereas regression coefficients reflect adjusted independent effects. The SHAP summary plot (**Figure 5B**) further illustrated the directional effects of each feature: lower BMI, prolonged PT, and male sex corresponded to positive SHAP values (red), significantly increasing the risk of sarcopenia. The dependency plots (**Figure 5C**) revealed an interactive effect between BMI and intervention: the protective effect of intervention (negative SHAP) was strengthened as BMI decreased, indicating that nutritional intervention is particularly beneficial for underweight patients The aggregation heatmap (**Figure 5D**) displayed the distribution of SHAP values at the population level, and the global decision plot (**Figure 5E**) demonstrated effective discrimination between sarcopenia patients (blue) and healthy individuals (red). At the individual level, taking patient #0 as an example, the local feature contribution plot (**Figure 5F**) and waterfall plot (**Figure 5G**) illustrate the contribution pathway of each variable. The local decision trajectory plot (**Figure 5H**) fully demonstrates the dynamic variation process of the patient’s logit value, providing traceable interpretive evidence for individualized risk assessment.

### 3.7 Subgroup analysis of model generalizability

**Figure 6:**
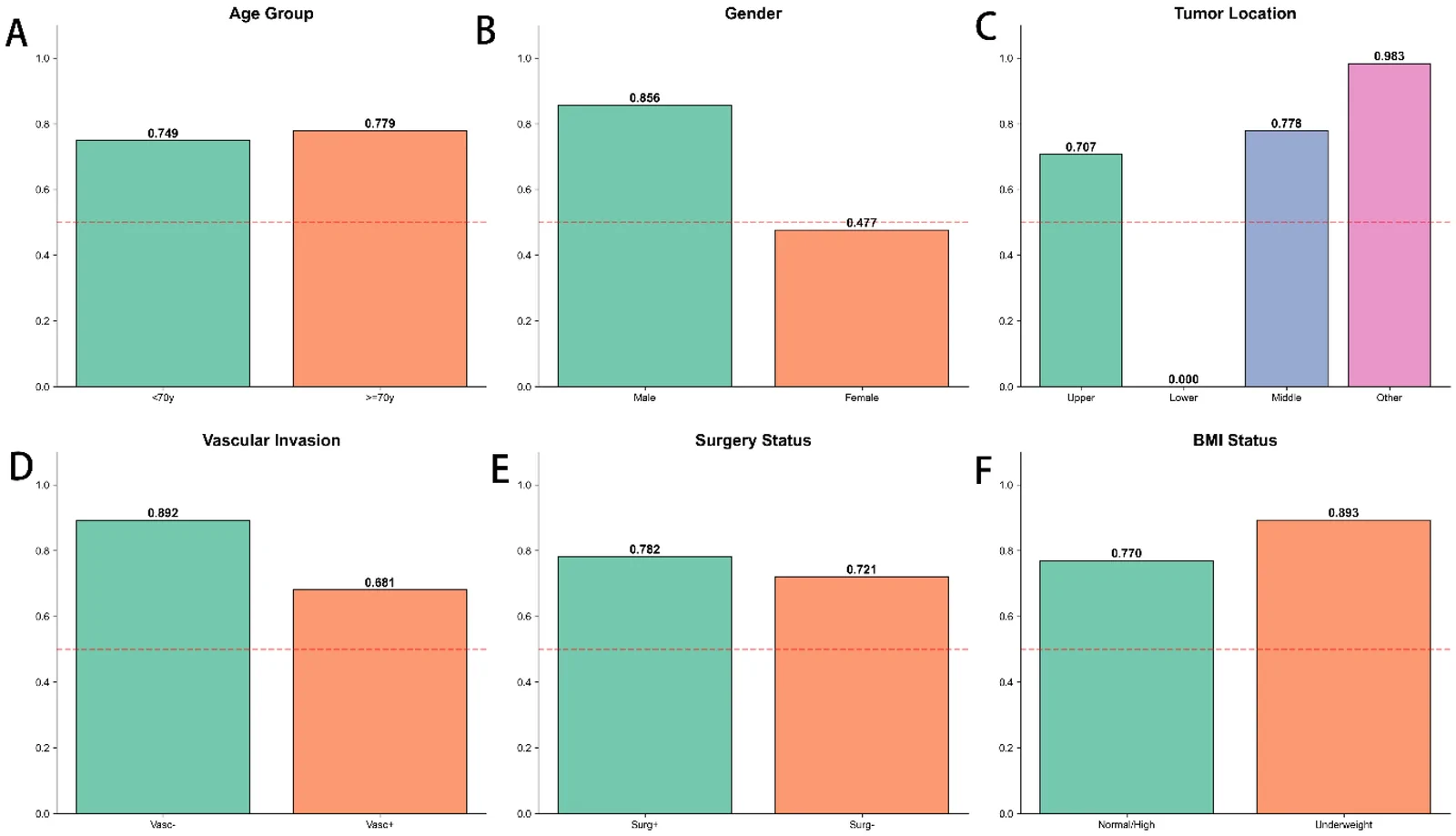
Predictive efficacy of the sarcopenia prediction model across clinical subgroups. **(A)** Age (<70 vs. ≥70 years). **(B)** Gender (male vs. female). **(C)** Tumor location (upper, lower, middle, and other). **(D)** Vascular invasion (Vasc− vs. Vasc+). **(E)** Surgery status (Surg+ vs. Surg−). **(F)** BMI status (normal/high vs. underweight). Dashed line indicates AUC = 0.50 reference.

Subgroup analyses were performed according to age, sex, tumor location, vascular invasion, surgical status, and BMI status (**Figure 6**). Overall, the model maintained favorable discriminatory ability in most subgroups. Model discrimination was comparable between patients aged <70 years and those aged ≥70 years (AUC: 0.749 vs. 0.779, respectively; **Figure 6A**). However, performance differed by sex, with a higher AUC in males than in females (0.856 vs. 0.477; **Figure 6B**). Across tumor-location subgroups, the AUCs were 0.707, 0.778, and 0.983 for upper, middle, and other locations, respectively; the AUC for the lower-location subgroup could not be reliably estimated (**Figure 6C**). The model also demonstrated better discrimination in patients without vascular invasion than in those with vascular invasion (AUC: 0.892 vs. 0.681; **Figure 6D**). The corresponding AUCs were 0.782 and 0.721 for patients with and without surgery, respectively (**Figure 6E**), and 0.770 and 0.893 for patients with normal/high BMI and underweight patients, respectively (**Figure 6F**). Collectively, the model performed particularly well in males, patients without vascular invasion, and underweight individuals, whereas relatively weak discriminatory performance was observed in the female subgroup.

Model discrimination was consistent across subgroups: age (0.749/0.779), sex (0.856/0.477), tumor location (0.797/0.778/0.983; lower excluded), vascular invasion (0.892/0.681), surgery (0.782/0.721), and BMI (0.770/0.893). The model performed particularly well in men, patients without vascular invasion, and underweight individuals, with a relatively weaker performance observed only in the female subgroup.

## 4 Discussion

Patients with esophageal cancer are often accompanied by severe malnutrition and sarcopenia due to dysphagia, mechanical obstruction, and high tumor metabolism. These conditions significantly reduce the patients’ tolerance to radiotherapy and chemotherapy as well as their survival prognosis (12). Currently, clinicians rely predominantly on computed tomography or magnetic resonance imaging to delineate the skeletal muscle index (SMI) when diagnosing sarcopenia (13, 14), Although high accuracy is achieved, drawbacks such as cumbersome operation, high dependence on equipment, and inability to realize rapid bedside assessment exist (12). Therefore, the exploration of a simple and comprehensive predictive model for sarcopenia in patients with esophageal cancer based on clinical baseline data and routine laboratory indicators is of great clinical significance (15, 16). This study sought to develop a clinical model for predicting sarcopenia in esophageal cancer, thereby supporting individualized early intervention.

To identify the independent influencing factors for sarcopenia, the patients were divided according to the score of the NRS-2002 scale (17, 18). Demographic characteristics, comorbidities, routine biochemical indicators, tumor stage, and other indicators of the patients were selected for univariate logistic regression analysis. Subsequently, significant variables were carried forward to multivariate logistic analysis. To screen more dimensional predictors and conduct a comprehensive and thorough prediction, this study further combined LASSO regression to screen independent predictors. Finally, by combining the predictors screened by the above-mentioned multiple methods, this study found that age, PT, history of diabetes, and cStage were independent influencing factors. Additionally, although age and sex did not emerge as statistically dominant in LASSO ranking, they represent indispensable demographic anchors in sarcopenia risk stratification. Age is a well-established non-modifiable determinant of muscle decline, and sex modulates both muscle mass distribution and hormonal milieu. Consequently, these two variables were incorporated into the final nomogram to ensure clinical generalizability and operational feasibility. The independent influencing factors may synergistically promote the occurrence of sarcopenia-related nutritional risk through multi-dimensional pathophysiological mechanisms. In elderly patients, the protein synthesis signaling pathway undergoes progressive decline, and advancing age attenuates IGF-1/PI3K/Akt/mTORC1 axis activity, which markedly blunts myocyte sensitivity to anabolic signals (19). Meanwhile, mitochondrial dysfunction and cumulative oxidative stress further exacerbate protein homeostasis imbalance (20, 21). Prolonged prothrombin time (PT) profoundly reflects impaired liver synthetic function and a state of systemic protein consumption (22, 23). As the main site for IGF-1 synthesis, the liver with impaired function leads to a decrease in circulating IGF-1 levels, which, in turn, inhibits mTORC1-mediated myoprotein synthesis. Prolonged PT reflects the interweaving of coagulation–fibrinolysis system activation and systemic inflammation, which inhibits Akt/mTOR activity through the TNF-α/IL-6-STAT3 pathway, forming a vicious cycle of “inhibition of protein synthesis to enhanced muscle decomposition” (24, 25). History of diabetes accelerates muscle attenuation through the dual mechanisms of insulin resistance and hyperglycemia (26). For patients diagnosed with type 2 diabetes, the activity of PI3K/Akt/mTOR pathway is reduced by 34%, the efficiency of protein synthesis is decreased by 30%–40%, and the inhibition of FoxO transcription factors is relieved, thereby upregulating the expression of muscle-specific E3 ubiquitin ligases (19). Chronic hyperglycemia further exacerbates myocyte damage through the WWP1-KLF15 pathway and the accumulation of advanced glycation end products (19, 27). Patients with advanced cStage have an increased risk of sarcopenia, which is closely associated with the systemic inflammatory response related to tumor burden (28, 29). Tumor progression drives the upregulation of pro-inflammatory mediators, including IL-6 and TNF-α, which inhibit muscle anabolism and promote protein decomposition through the JAK/STAT3 pathway (1). The above-mentioned findings suggest that clinicians should initiate functional assessment and intervention for sarcopenia as early as possible in esophageal cancer patients who are elderly, in advanced stage, and complicated with metabolic dysfunction.

Although body mass index (BMI) is a traditional indicator to evaluate nutritional status, it is often normal or even high in patients with “sarcopenic obesity”, showing a certain degree of deception (30, 31). This study used the SHAP method to analyze the contribution of each indicator and found that, in addition to BMI, PT and cStage contributed the most to the predictive ability. PT was positively correlated with the risk of sarcopenia, reflecting the association between decreased liver synthetic capacity and muscle loss. This finding is consistent with studies on sarcopenia associated with liver cancer: hepatic parenchymal damage leads to reduced IGF-1 synthesis, which, in turn, inhibits the mTORC1 pathway and ultimately impairs muscle regeneration ability (32). This combination of multiple indicators makes up for the defect that a single BMI cannot accurately reflect muscle mass, which is consistent with the current multi-dimensional assessment strategy (1), suggesting that biochemical indicators have important supplementary value in the diagnosis of sarcopenia.

To evaluate the diagnostic efficacy for sarcopenia, the model demonstrated favorable discrimination. Wang Y et al. constructed a model in esophageal cancer based on CT radiomics (32) with an AUC of 0.87, while Kim et al. (33) developed a multi-modal model integrating clinical and imaging features in gastric cancer patients, with an AUC of 0.845. Notably, the present model achieves similar efficacy relying only on routine clinical indicators, which confers better clinical accessibility. Compared with a single indicator, the predictive model constructed in this study, by combining multi-dimensional indicators, has higher sensitivity and specificity. Previous studies have confirmed that the AUC of a single BMI for predicting sarcopenia is only 0.65–0.70, while the multi-indicator combined model can be improved to more than 0.80 (34, 35). Calibration and decision curves suggest that the model may provide net benefit for low-cost triage, especially when CT segmentation, grip strength, or gait-speed testing is unavailable (19, 36). Subgroup analyses demonstrated consistent performance across most clinical strata; however, the female subgroup exhibited suboptimal discrimination (AUC 0.477), likely reflecting the small stratum (*n* = 45) with limited positive events causing statistical instability, compounded by sex-specific differences in muscle mass and hormonal regulation (e.g., estrogen-mediated myoprotection). Sex-balanced validation is warranted before clinical application in female patients. Compared with existing screening tools, SARC-F exhibits low sensitivity (21%–46%) and relies on subjective self-report (37), whereas SARC-F-EBM and SARC-CalF improve the accuracy but still require patient cooperation and trained staff. Our model requires only routine admission variables without additional cost or patient exertion, enabling immediate pre-treatment risk stratification in esophageal cancer patients scheduled for neoadjuvant therapy or surgery (38).

This study is not without limitations. Its reliance on a single-center, retrospective cohort restricts broader applicability, which may lead to selection bias. In the future, it is planned to further carry out multi-center prospective cohort studies to verify the external stability of the model. Furthermore, the retrospective design necessitated NRS-2002 ≥3 as a surrogate for sarcopenia-related nutritional risk, pending prospective validation against definitive EWGSOP/AWGS criteria (CT-SMI, grip strength, and gait speed). Therefore, the outcome predicted by this model should be interpreted strictly as sarcopenia-related nutritional risk rather than clinically confirmed sarcopenia, and the model should not replace standard assessments of muscle mass, muscle strength, or physical performance. Additionally, relying on the R Shiny online interactive platform (Web-based Calculator) developed in this study, the application potential of this tool in elderly rehabilitation monitoring, remote nutritional intervention, and full-course digital management of sarcopenia will be explored so as to promote the clinical implementation of precise screening and individualized intervention (39, 40).

In summary, the predictive model based on multi-dimensional indicators developed in this study can effectively stratify sarcopenia risk in patients with esophageal cancer. This model not only has good predictive accuracy, but also the included indicators are easy to obtain. Combined with the interactive online decision-making tool, it has strong clinical practical value and is expected to be further promoted and applied in clinical practice. Nevertheless, its clinical application should be limited to preliminary screening for sarcopenia-related nutritional risk, and patients identified as high risk require further confirmation using established AWGS/EWGSOP diagnostic criteria.

## Data Availability

The raw data supporting the conclusions of this article will be made available by the authors, without undue reservation.
