# Supplementary material for: A LASSO-based nomogram for predicting sarcopenia-related nutritional risk in esophageal cancer patients: model development, validation, and an interactive clinical decision tool
Source: Front Oncol. 2026 Sep 1;16:1863123. doi: 10.3389/fonc.2026.1863123 (PMC13574675; doi:10.3389/fonc.2026.1863123)
Supplement: Supplementary file 1 [file Table1.docx]

# Supplementary Table S1

***Full Logistic Regression Model Coefficients, Equation, and Variable Coding***

## Variable Coding Scheme

| **Variable** | **Type** | **Coding / Units** |
| --- | --- | --- |
| Age | Continuous | Years |
| Sex | Binary | 0 = Female; 1 = Male |
| BMI | Continuous | kg/m² |
| PT | Continuous | Seconds |
| Diabetes | Binary | 0 = No; 1 = Yes |
| Intervention | Binary | 0 = No; 1 = Yes |
| cStage | Categorical (Reference: cStage I–II) | Dummy variables: • cStage_Other: 1 = Other specified stages, 0 = Reference • cStage_IV: 1 = Stage IV, 0 = Reference • cStage_Unknown: 1 = Unknown stage, 0 = Reference |

## Logistic Regression Model Equation

**Linear Predictor (LP):**

LP = β₀ + β₁×Age + β₂×Sex + β₃×BMI + β₄×PT + β₅×Diabetes + β₆×Intervention + β₇×cStage_Other + β₈×cStage_IV + β₉×cStage_Unknown

**Predicted Probability:**

P (Sarcopenia-Related Nutritional Risk) = 1 / (1 + e^(−LP))

## Model Coefficients and Performance Metrics

| **Parameter** | **β (Coefficient)** | **Standard Error** | **Odds Ratio** | **95% CI** | **P-value** |
| --- | --- | --- | --- | --- | --- |
| Intercept | −4.18 | 0.82 | — | — | <0.001 |
| Age | 0.095 | 0.044 | 1.10 | 1.00–1.22 | 0.030 |
| Sex (Male vs. Female) | 0.62 | 0.31 | 1.86 | 1.01–3.42 | 0.045 |
| BMI | −0.11 | 0.055 | 0.90 | 0.81–1.00 | 0.048 |
| PT | 0.46 | 0.20 | 1.59 | 1.07–2.72 | 0.024 |
| Diabetes (Yes vs. No) | 0.80 | 0.37 | 2.23 | 1.08–4.60 | 0.031 |
| Intervention (Yes vs. No) | −1.02 | 0.34 | 0.36 | 0.18–0.70 | 0.003 |
| cStage_Other (vs. I–II) | 0.53 | 0.27 | 1.70 | 0.99–2.91 | 0.052 |
| cStage_IV (vs. I–II) | 0.75 | 0.32 | 2.12 | 1.13–3.97 | 0.020 |
| cStage_Unknown (vs. I–II) | 0.40 | 0.29 | 1.49 | 0.84–2.64 | 0.168 |

*Note: Coefficients β were linearly transformed to a 0–100 point scale for nomogram construction. The total nomogram score was converted to predicted probability using the logistic function above.*

## Missing Data and Computational Details

- Missing data handling: Variables with >5% missing values were excluded. Remaining missing values were imputed using multiple imputation by chained equations (MICE, 10 iterations) under the missing-at-random assumption.
- Software: R version 4.3.1; packages: glmnet (v4.1-7), pROC (v1.18.5), rms (v6.7-1), mice (v3.16.0), and reticulate (v1.35.0) for Python SHAP integration.
- Online tool: "Sarcopenia Intelligence: Clinical Prediction & Interpretation System" (v1.0), developed with R Shiny (v1.8.0). URL: https://ytky622.shinyapps.io/Sarcopenia_Predictor_2026/
